# Finite element analysis of medial closing and lateral opening wedge osteotomies of the distal femur in relation to hinge fractures

**DOI:** 10.1186/s40634-023-00597-w

**Published:** 2023-03-27

**Authors:** Michel Meisterhans, Andreas Flury, Christoph Zindel, Stefan M. Zimmermann, Lazaros Vlachopoulos, Jess G. Snedeker, Sandro F. Fucentese

**Affiliations:** 1 Department of Orthopedics, Balgrist University Hospital, University of Zurich, Zurich, Switzerland; 2 Institute for Biomechanics, ETH Zurich, Zurich, Switzerland

**Keywords:** Distal femur osteotomies, Medial closed wedge, Lateral open wedge, Finite element analysis, Statistical shape model, Hinge fracture risk, Biomechanics

## Abstract

**Purpose:**

Intraoperative hinge fractures in distal femur osteotomies represent a risk factor for loss of alignment and non-union. Using finite element analysis, the goal of this study was to investigate the influence of different hinge widths and osteotomy corrections on hinge fractures in medial closed-wedge and lateral open-wedge distal femur osteotomies.

**Methods:**

The hinge was located at the proximal margin of adductor tubercle for biplanar lateral open-wedge and at the upper border of the lateral femoral condyle for biplanar medial closed-wedge distal femur osteotomies, corresponding to optimal hinge positions described in literature. Different hinge widths (5, 7.5, 10 mm) were created and the osteotomy correction was opened/closed by 5, 7.5 and 10 mm. Tensile and compressive strain of the hinge was determined in a finite element analysis and compared to the ultimate strain of cortical bone to assess the hinge fracture risk.

**Results:**

Doubling the correction from 5 to 10 mm increased mean tensile and compressive strain by 50% for lateral open-wedge and 48% for medial closed-wedge osteotomies. A hinge width of 10 mm versus 5 mm showed increased strain in the hinge region of 61% for lateral open-wedge and 32% for medial closed-wedge osteotomies. Medial closed-wedge recorded a higher fracture risk compared to lateral open-wedge osteotomies due to a larger hinge cross-section area (60–67%) for all tested configurations. In case of a 5 mm hinge, medial closed-wedge recorded 71% higher strain in the hinge region compared to lateral open-wedge osteotomies.

**Conclusion:**

Due to morphological features of the medial femoral condyle, finite element analysis suggests that lateral-open wedge osteotomies are the preferable option if larger corrections are intended, as a thicker hinge can remain without an increased hinge fracture risk.

## Introduction

Genu valgum deformity is less common than varus malalignment, but no less relevant concerning unicompartmental joint wear [5, 8, 10, 38]. Moreover, valgus malalignment is associated with patellofemoral maltracking [9, 13, 14, 16, 32]. A hypoplastic lateral condyle of the femur is the most common cause for idiopathic valgus malalignment. The distal femoral osteotomy (DFO) is the preferable surgical approach therefore [45]. Hereby, a parallel joint line can be achieved by either a medial closed wedge (MCW) or lateral open wedge (LOW) technique [45, 46]. Independent of the technique used, DFO to treat lateral compartment disease was shown to improve clinical scores with good mid and long-term survivorship [5, 8, 24]. The most commonly reported drawbacks include a high rate of complications (9%) and a considerable number of delayed (4%) or non-unions (3%), in a systematic review with 372 DFOs, respectively [45]. The main reason cited for these complications is a fracture of the hinge, which leads to reduced axial and torsional stability, and therefore increased movement and stress across the osteotomy gap and at the bone-implant construct [2, 23, 35, 44, 45]. According to the literature, cortical hinge fractures occur in 43—57% of cases, slightly more frequent in open-wedge osteotomies [15, 18, 37, 44].

Previous research identified larger osteotomy gaps and a hinge location close to the opposite cortex as risk factors associated with unstable cortical hinge fractures [26, 27, 37, 44]. Moreover, based on bone density and soft tissue coverage, optimal hinge positions have been suggested [23, 44]. The pathomechanism of hinge fractures, however, is not yet fully understood. So far, biomechanical studies focused on modified joint loading forces [1, 47] or osteosynthesis material [36, 41, 43]. To this date, no biomechanical study exists, however, that systematically assessed risk factors of hinge fractures in varus creating DFOs.

Using Finite Element (FE) analysis, the goal of this study was to investigate previously-described parameters that are associated with hinge fractures. The hypothesis was that 1) a larger osteotomy gap and a larger hinge width increase the risk of intraoperative hinge fractures in DFO, as does 2) a higher cortical bone density of the hinge. Moreover, it was hypothesized that 3) there is a higher risk of hinge fractures in MCW compared to LOW.

## Methods

### Development of DFO models

**Fig. 1:**
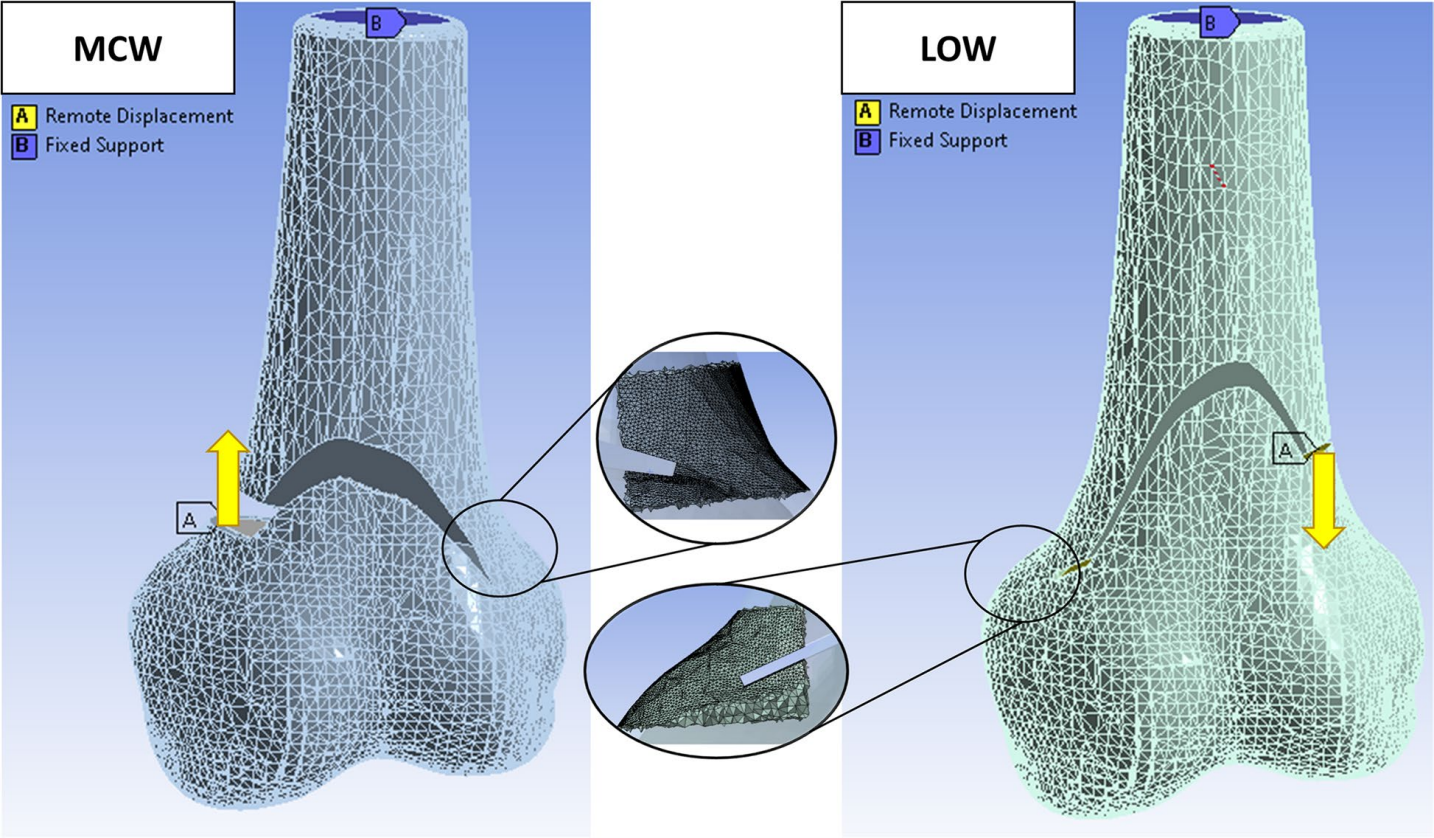
Osteotomy configurations and loading conditions for MCW and LOW, fixed support (**B**) at the proximal end of the segmented femur and remote displacement (**A**). Higher meshed hinge area of cortical bone in scope from a posterior view. Mean compressive and tensile principal strain was read out for this region. MCW: Medial closed wedge; LOW: Lateral open wedge

Based on 61 femora from routine postmortem CT scans, a previously published statistical shape model of the human femora was used [12]. To create this model, a non-rigid registration algorithm [29] was used and 20 principal components were defined to represent 99% of the shape variance [12]. Using Autodesk Inventor (Autodesk Inc., San Rafael, USA), biplanar DFO were created with a horizontal osteotomy angle of 25° for MCW and 18° for LOW, respectively (Fig. 1), with a vertical osteotomy angle of 70° [35]. The coronal plane was defined by a plane intersecting the medial and lateral condyle and the greater trochanter and was used as a reference for the horizontal osteotomy angle. The hinge axis was orientated orthogonal to the coronal plane, corresponding to the sagittal plane. Hinge location was defined according to most recent literature at the proximal margin of the adductor tubercle for LOW [44], and at the upper border of the lateral femoral condyle for MCW [23], respectively. After performed DFO, the femur was sectioned 200 mm proximal to the joint line to reduce computational effort (see Fig. 1).

### Simulated factors of interest

To investigate the influence of correction quantity on hinge fractures, wedge sizes of 5 mm, 7.5 mm, and 10 mm were simulated (Fig. 1). In case of a closing-wedge osteotomy, the gap was defined as 1.5 mm respecting the saw blade thickness. Next, hinge widths of 5 mm, 7.5 mm, and 10 mm were imitated, reflecting the range of hinge width in current DFO techniques [44]. From the outer surface of the statistical femora shape model a volume element was created with a constant thickness for the entire model to represent cortical bone, the remaining inner volume from the statistical shape model was considered cancellous bone. Two variables for cortical thickness were investigated: 3 mm (physiological) and 1.5 mm (pathological/osteoporotic) [6, 19].

### Finite element analysis and material properties

For FE analysis, Ansys Workbench (Version R1, Ansys Inc., Canonsburg, USA) was used to create a mesh using tetragonal elements with an approximate element size of 1 mm and 0.5 mm in the hinge area. A convergence analysis was performed and an aspect ratio of < 4 was recorded for 95% of its elements. The analysis was performed without the use of a bone-void filler due to the following reasons [17, 22]: 1) maximal size of the osteotomy gap was set to 10 mm, and 2) to simulate the worst-case scenario for hinge loading. Cortical bone was considered transversely isotropic (Elastic Modulus (E): Ex = Ey = 11.5 GPa, Ez = 17 GPa; Poisson's ratio (v): vxy = 0.51, vxz = vyz = 0.31 GPa), whereas cancellous bone was modeled as a linear isotropic material property (E = 2.13 GPa and v = 0.3) [21].

### Loading and boundary conditions

Contrary to the femur condyles, the sectioned femur shaft was considered fully constrained, simulating the patient lying on his back during the procedure. Therefore, the proximal femur remained rigid and the distal osteotomy surface was distracted (LOW) with a remote displacement vector in axial direction (5, 7.5 and 10 mm) at the border of the lateral distal osteotomy surface. For MCW, the distal osteotomy surface was reduced by applying a remote displacement vector in axial direction (5, 7.5 and 10 mm) on the medial condyle, in line with the medial distal osteotomy surface (Fig. 1).

### Outcome measures

A hinge region was defined as the area of interest, corresponding to the area where hinge fractures usually occur [42] (Fig. 1). The mesh element number for the cortical bone volume showed a congruence of 96% averaged for all hinge widths (Table 1). Higher stress and strain in the cortical than the cancellous bone is expected: cortical bone has a higher Young's modulus and consequently carries more load on wedge opening or closing. Though cancellous bone responses are included in this study, the prevention of unwanted cortical bone fracture is of greatest interest and thus cortical bone was the main focus. Because bone fractures are strain-determined [31], primary outcome measure was the cortical bone maximum principal strain and minimum principal strain in the hinge area. According to the crack tip stress theory [7], for an infinitesimally fine crack tip (zero radius) wedge opening would cause stress at the apex of the crack tip to approach infinity. Crack tip proximity can give falsely elevated peak stresses and strains, therefore, the mean principal strain in the hinge region was selected as an outcome measure in preference. In order to establish hinge fracture potential, a risk for fracture (RF) was calculated. RF was defined as the ratio between either mean tensile or mean compressive strain in the hinge region and the corresponding ultimate strain [3, 39]:

$$RF={\varepsilon }_{max}/{\varepsilon }_{lim}$$

ε_max_: is the mean tensile/compressive strain in the hinge region

ε_lim_: is the ultimate strain. The ultimate strain for bone is different under compressive and tensile conditions.

**Table 1:** Element mesh number for hinge area for cortical bone (3 mm cortical thickness configuration)

Mesh element number hinge region			
Hinge width [mm]	**MCW**	**LOW**	**LOW/MCW [%]**
5	43,744	41,173	94
7.5	44,033	42,521	97
10	44,212	42,559	96

*LOW* Lateral open wedge, *MCW* Medial closed wedge

According to Bayraktar et al. [3], the ultimate compressive strain is ε_lim-compressive_ = 0.0104, whereas the ultimate tensile strain was 70% of that value (ε_lim-tensile_ = 0.0073). RF values greater than 1 indicated a certain fracture and RF values lower than 1 indicated no fracture occurrence.

## Results

### Amount of correction and cortical thickness

**Fig. 2:**
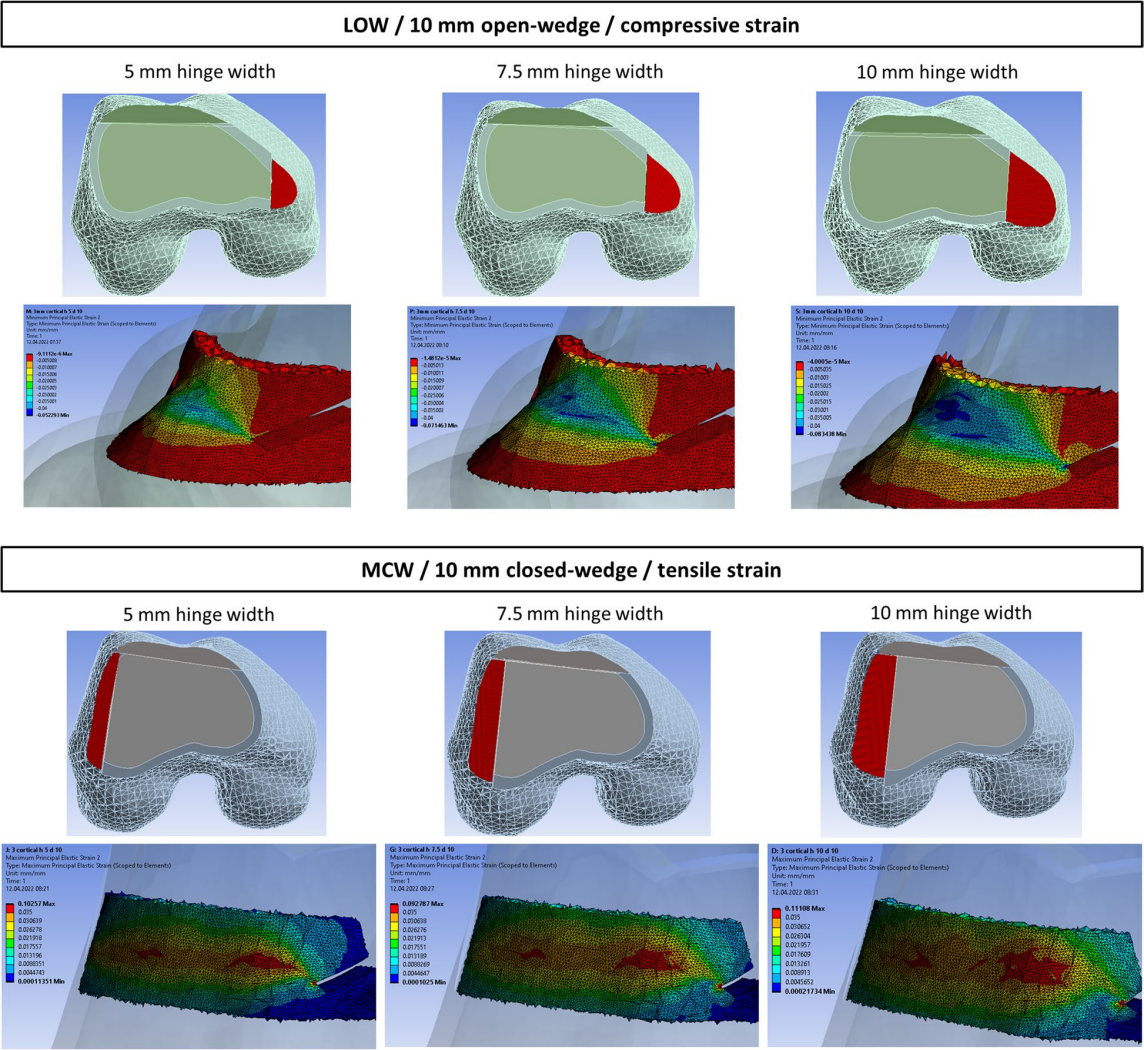
LOW (upper figure) and MCW (lower figure) with different hinge widths of 5, 7.5, and 10 mm. The cross-section area of the hinge is visible in the upper row. Lower row shows strain distribution in the hinge area. LOW: maximum (blue) and minimum (red) principle compressive strain. MCW: maximum (red) and minimum (blue) principle tensile strain. MCW: Medial closed wedge; LOW: Lateral open wedge

**Fig. 3:**
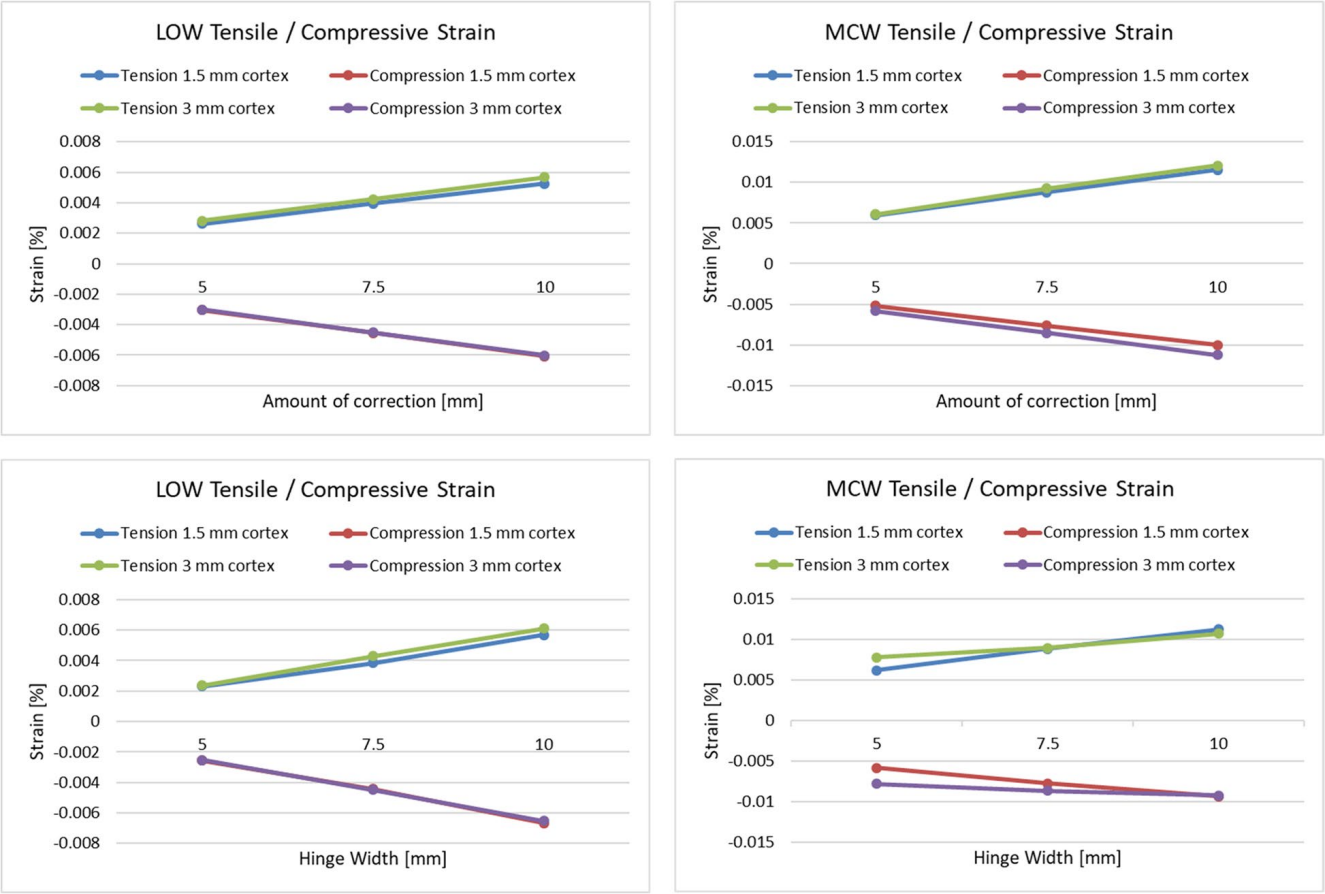
Upper figures: Linear correlation between strain increase (%) and amount of correction for LOW (left) and MCW (right). No difference is shown between different cortical thicknesses (1.5 versus 3 mm). Lower figures: Tensile and compressive strain (%) plotted for different hinge widths for LOW (left) and MCW (right), for cortical thickness of 1.5 and 3 mm, and averaged for all correction amounts. MCW: Medial closed wedge; LOW: Lateral open wedge

For LOW (Fig. 2), there was compressive strain at the outer cortex, with maximal compressive strain proximal to the hinge. Tensile strain occurred at the inner cortex. The behavior of strain was reversed in case of MCW (Fig. 2). The larger the correction, the larger the strain. In LOW, doubling the correction from 5 to 10 mm increased mean tensile and compressive strain by 50% (Table 2). In MCW the mean tensile and compressive strain increased by 48% for a 10 mm instead of a 5 mm closed-wedge (Table 2). The linear correlation is shown in Fig. 3.

**Table 2:** Change in mean tensile and compressive strain for different correction amounts and different hinge widths

		**amount of correction [mm]**		
		**5 to 7.5**	**7.5 to 10**	**5 to 10**
**LOW**	change in strain [%]	34	25	50
**MCW**	change in strain [%]	32	24	48
		**hinge width [mm]**		
		**5 to 7.5**	**7.5 to 10**	**5 to 10**
**LOW**	change in strain [%]	42	32	61
**MCW**	change in strain [%]	19	16	32

*LOW* Lateral open wedge, *MCW* Medial closed wedge

A 3 mm compared to a 1.5 mm cortical thickness recorded a 4% and 8% higher tensile and compressive strain in LOW and MCW, respectively.

### Hinge width

A 10 mm hinge width produced the largest mean tensile and compressive strain for LOW and MCW (Fig. 3), because the strain is experienced over a larger volume of bone (Fig. 2 and 5). In LOW, a hinge of 10 mm recorded 61% higher mean strain compared to a hinge width of 5 mm (Table 2). In MCW, a hinge of 10 mm compared to 5 mm recorded 32% higher mean strain respectively (Table 2).

### Strain in LOW versus MCW

Comparing LOW and MCW with a 5 mm hinge regarding strain encountered in the hinge region (Table 3), MCW recorded 71% higher mean strain. Hinge width and strain difference was inversely proportional, so that strain difference between MCW and LOW declined with wider hinges. Thus, strain was only 40% higher in MCW compared to LOW in case of a 10 mm hinge (Table 3).

**Table 3:** Mean tensile and compressive strain for different hinge widths, averaged for all correction amounts

LOW vs MCW	hinge width [mm]		
	**5**	**7.5**	**10**
LOW mean tensile and compressive strain [%]	0.002	0.004	0.006
MCW mean tensile and compressive strain [%]	0.007	0.009	0.010
**change in strain LOW/MCW [%]**	29	44	60

Positive values mean increased strain values for MCW compared to LOW

*LOW* Lateral open wedge, *MCW* Medial closed wedge

### Fracture risk

In LOW, a hinge fracture was recorded only in case of a 10 mm hinge in combination with a 10 mm open-wedge correction for tensile strain on the inner cortex (Table 4). In MCW, a RF > 1 was calculated for each simulation (Table 4). Independent of the hinge width, every scenario with a 10 mm closed-wedge correction showed an RF of > 1. For a 7.5 mm or 5 mm correction, the cortical bone failed if the hinge was 7.5 mm or more (Table 4).

**Table 4:**
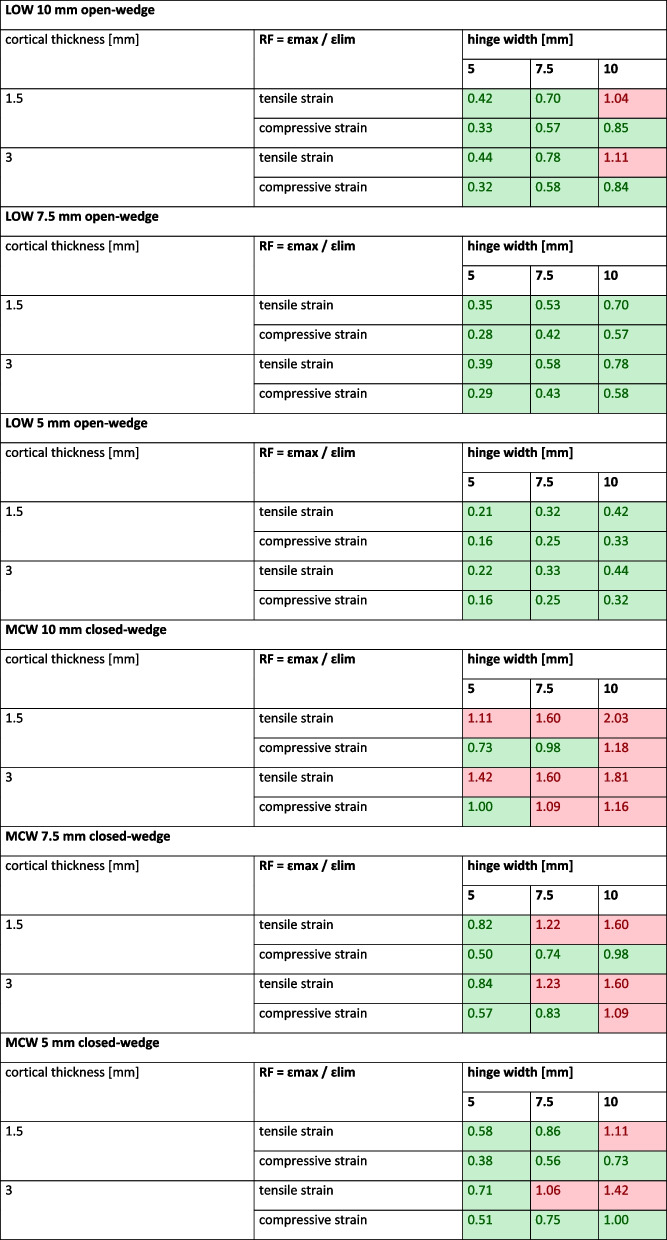
Fracture risk for LOW and MCW depending on amount of correction

*LOW* Lateral open wedge, *MCW* Medial closed wedge, *RF* Risk for fracture, values greater than 1 stated in red indicate a hinge fracture, values smaller than 1 stated in green indicate an intact hinge

### Synopsis

Due to the morphology of the femur, the cross-section area of the hinge is significantly bigger in MCW compared to LOW (Table 5). This results in a higher risk of hinge fractures in MCW, especially in case of larger corrections. If a LOW is performed with the goal of 10 mm correction, the hinge should not exceed a thickness of 7.5 mm. For MCW, however, the hinge needs to be thinned out more extensively, since a 7.5 mm closed-wedge correction with a 5 mm hinge was the only scenario where no hinge fracture occurred.

**Table 5:** Hinge cross-section for cortical bone and total bone (cortical and cancellous)

Cross-section area [mm^2^]						
	**MCW**		**LOW**		**change [%]**	
**Hinge width [mm]**	cortical	total	cortical	total	cortical (LOW/MCW)	total (LOW/MCW)
**5**	131	174	56	57	43	33
**7.5**	149	284	92	104	62	37
**10**	162	402	117	160	72	40

*LOW* Lateral open wedge, *MCW* Medial closed wedge

## Discussion

The most important findings of this study were that a higher correction amount as well as a larger hinge width increase the risk for a hinge fracture in DFO. Moreover, due to morphological features of the medial femoral condyle, the fracture risk is higher in MCW compared to LOW.

The hinge fracture being an accepted risk factor for malunion and loss of correction after DFO [15, 18], substantial efforts have been made to mitigate the risk of unstable cortical hinge fractures in DFO [23, 37, 44]. This study is in accordance with recent literature where technical and constitutional risk factors were investigated and larger osteotomy gaps were associated with an increased risk of hinge fractures [26, 30, 34, 37, 44]. However, different results were reported regarding the role of hinge width. In HTO, Ogawa et al. [34] and Nakamura et al. [30] showed that a deep osteotomy involving both anterior and posterior cortices reduces the risk of a hinge fracture compared to a shallow osteotomy. Brinkman et al. [4] suggested an optimal hinge width of 10 mm. In LOW DFO, a mean hinge width of 8.8 ± 4.2 mm showed significantly less hinge fractures compared to 7.2 ± 5.1 mm in the study of Winkler et al. [44]. Nonetheless, this finding could not be confirmed for lateral closing-wedge DFO by the same study group [37]. From a biomechanical point of view however, the hinge should be less than the currently established 10 mm. According to the newly gained insights provided by this study, a remaining hinge of 5 mm records the lowest amount of strain and thus carries the lowest risk of fracture. Furthermore, a smaller hinge requires less force to apply the correction according to Hooke's law and makes the procedure more controllable. The elastic deformation of the hinge can be described by the Hooke's law and is inversely proportional to the elastic modulus of cortical bone and the hinge-cross section, thus also depending on the cortical thickness of the hinge region. This study recorded a 4% respectively 8% tensile and compressive strain decrease for 1.5 mm instead of 3 mm cortical thickness in the hinge region. Kim et al. [23] proposed an ideal lateral hinge position for MCW at the upper edge of the lateral condyle with the rational of not only having stable soft tissue coverage but also low cortical bone density and therefore increased capacity of deformation. These findings support the theory of aiming for hinge positions with low cortex thickness or low cortical density to reduce the fracture risk.

However, this model should not be applied to infinity by creating a remaining hinge as small as possible. At some point the remaining hinge will be so small that a microcrack created during sawing or correction will fracture the hinge, which might be a possible explanation for the above-mentioned findings in clinical studies. Such microcrack occurs at the apex of the hinge during DFO and the bone resists such stress-risers to much greater extent than anticipated on the basis of its elastic or elastoplastic properties [11, 25]. However, no method is currently available to achieve a balance between creating a more favorable strain environment and preserving cortical bone stock to minimize hinge fracture risk. Further cadaveric studies are needed to assess the relation of a thin remaining hinge and possible microcrack propagation.

Several studies have been performed to find the best approach for varus-producing DFO. MCW has the advantages of direct bony contact, which leads to inherent stability and reliable bony healing compared to the need for potential bone grafting in large LOW corrections [46]. However, nonunion rates of LOW do not appear to be inferior to MCW [28, 45]. Hardware irritation is less frequently reported in MCW compared to LOW, where the plate is placed directly underneath the iliotibial band, leading to a higher hardware removal rate [20, 45]. LOW, on the other hand, allows correction and adjustment of distraction in order to optimize the mechanical leg axis, and places the plate on the mechanically preferred tension side [46]. Nevertheless, according to the new insights found in this study, several additional mechanical properties must be assigned to either of two DFO options. Due to the morphology of the distal femur, which is responsible for larger remaining hinge cross-section area in MCW compared to LOW osteotomies, MCW bears a higher risk of hinge fractures. In detail, there was a higher risk of fracture for all tested MCW configurations. Therefore, LOW seems to be the preferable DFO if larger corrections are intended, as a thicker hinge can remain without increasing the risk of a hinge fracture. If MCW is performed, due to the potential advantage of direct bony contact, a smaller hinge width than the currently established 10 mm should be achieved.

Overall, the findings of this study do not allow prediction whether a fractured hinge in LOW or MCW is more prone to instability. Due to the amount of remaining hinge material in MCW, a fracture might not propagate through the entire hinge and be more stable. This FE study focused on hinge fractures, but not on their extension, dislocation or potential correction loss. Figure 2 allows us to hypothesize that the hinge fracture in LOW resulting from tensile strain at the inner cortex spreads to the area of maximal compressive strain, which is proximal to the hinge. However, according to Winkler et al. [44], only 20% of hinge fractures seem to extend proximally (referred to as type 3 hinge fractures).

This study has some limitations. A femur statistical shape model with a constant cortical thickness was used that does not reflect the graduated trabecular structure of distal femur cancellous bone. Nonetheless, an anisotropic heterogeneous bone was modeled using isotropic and homogenous material properties and a linearly elastic analysis was performed, making the results applicable to the statistical shape femora model. This is a common method and does not discredit the differences found between geometries of both osteotomies [40]. A further limitation of this study is the fact that surrounding soft tissue, including tendons and muscles, was neglected. This might affect the findings, since some studies have suggested predestined hinge locations based on soft tissue coverage to reduce the risk of hinge fracture [23, 33]. Furthermore, the simulated load conditions reflect only the intraoperative situation of MCW and LOW DFO before plating.

## Conclusion

LOW demonstrates a lower intraoperative hinge fracture risk compared to MCW in varus-producing DFO due to the morphological features of the medial femoral condyle. Therefore, LOW seems to be the preferable option if larger corrections are intended, as a thicker hinge can remain without increasing the risk of a hinge fracture. If MCW is performed, the currently established hinge width of approximately 10 mm should be reduced. Due to the limitation of the study regarding microfracture propagation a recommendation regarding the ideal hinge width in clinical use can not be given.
